# Erythropoietin Mediated Bone Loss in Mice Is Dose-Dependent and Mostly Irreversible

**DOI:** 10.3390/ijms21113817

**Published:** 2020-05-27

**Authors:** Albert Kolomansky, Sahar Hiram-Bab, Nathalie Ben-Califa, Tamar Liron, Naamit Deshet-Unger, Moshe Mittelman, Howard S. Oster, Martina Rauner, Ben Wielockx, Drorit Neumann, Yankel Gabet

**Affiliations:** 1 Department of Cell and Developmental Biology, Sackler Faculty of Medicine, Tel Aviv University, 6997801 Tel Aviv, Israel; alexkolomansky228@gmail.com (A.K.); alloulnat@gmail.com (N.B.-C.); naamitd@yahoo.com (N.D.-U.); 2 Department of Medicine A, Tel Aviv Sourasky Medical Center, Sackler Faculty of Medicine, Tel Aviv University, 6423906 Tel Aviv, Israel; moshemt@gmail.com (M.M.); howardo@tlvmc.gov.il (H.S.O.); 3 Department of Anatomy and Anthropology, Sackler Faculty of Medicine, Tel Aviv University, 6997801 Tel Aviv, Israel; saharurit@gmail.com (S.H.-B.); tamarlrn@gmail.com (T.L.); 4 Department of Medicine III, Dresden University Medical Center, 01307 Dresden, Germany; Martina.Rauner@uniklinikum-dresden.de; 5 Institute for Clinical Chemistry and Laboratory Medicine, Technische Universität Dresden, 01307 Dresden, Germany; ben.wielockx@tu-dresden.de

**Keywords:** erythropoietin, bone, osteoclasts, anemia

## Abstract

Recent studies have demonstrated that erythropoietin (EPO) treatment in mice results in trabecular bone loss. Here, we investigated the dose-response relationship between EPO, hemoglobin (Hgb) and bone loss and examined the reversibility of EPO-induced damage. Increasing doses of EPO over two weeks led to a dose-dependent increase in Hgb in young female mice, accompanied by a disproportionate decrease in trabecular bone mass measured by micro-CT (µCT). Namely, increasing EPO from 24 to 540 IU/week produced a modest 12% rise in Hgb (20.2 ± 1.3 mg/dL vs 22.7 ± 1.3 mg/dL), while trabecular bone volume fraction (BV/TV) in the distal femur decreased dramatically (27 ± 8.5% vs 53 ± 10.2% bone loss). To explore the long-term skeletal effects of EPO, we treated mice for two weeks (540 IU/week) and monitored bone mass changes after treatment cessation. Six weeks post-treatment, there was only a partial recovery of the trabecular microarchitecture in the femur and vertebra. EPO-induced bone loss is therefore dose-dependent and mostly irreversible at doses that offer only a minor advantage in the treatment of anemia. Because patients requiring EPO therapy are often prone to osteoporosis, our data advocate for using the lowest effective EPO dose for the shortest period of time to decrease thromboembolic complications and minimize the adverse skeletal outcome.

## 1. Introduction

Erythropoietin (EPO) is a glycoprotein hormone mainly expressed by peritubular fibroblasts in the renal cortex. EPO secretion is regulated by hemoglobin (Hgb) levels and tissue oxygenation (pO_2_) in the outer medulla of the kidney. The synthesis of EPO is regulated at the transcriptional level, i.e., a low tissue pO_2_ leads to up-regulation of EPO synthesis and consequent stimulation of erythrocyte production. Erythropoiesis is a slow-acting process, following a rise in plasma EPO, it takes 3–4 days before reticulocytes (immature red cells) become apparent [1,2,3]. 

The normal concentration of EPO in rat plasma is about 25 U/l. After acute hypoxic stress, the plasma EPO concentration rises within 1.5–2 hours and can increase up to 100-fold within 18 h [4].

Recombinant human EPO (rHuEPO) is used in clinical practice for the treatment of several types of anemia [3,5,6]. It has been long recognized by clinicians that, while low Hgb levels lead to anemia, excessive Hgb levels can increase mortality as a result of cardiovascular and thromboembolic events. Hence, the regulation of EPO dosing is a major concern and effective mathematical methods are being developed to determine the appropriate dose of EPO to maintain the target Hgb levels [7,8]. 

Unrelated to the primary erythropoietic function, EPO-R is expressed in diverse tissues including bone cells [9,10]. In bone, osteoclasts are specialized bone resorbing cells derived from monocytes, where monocyte differentiation into osteoclasts is driven by the receptor activator for the nuclear factor κB (RANK)/RANK ligand (RANKL)/osteoprotegerin system and M-CSF [11]. Osteoblasts, derived from mesenchymal stem cells, are responsible for bone formation [12]. Previous studies have demonstrated that both osteoblasts and osteoclasts express EPO-R. Regarding the latter, the reported literature is somewhat controversial, but indeed, it may merely reflect the fact that EPO-R expression in osteoblasts is detected only during specific differentiation stages [13]. In addition, detecting the expression of EPO-R at the protein level is challenging due to the lack of a specific antibody [14,15]. Nevertheless, EPO both increases bone resorption and decreases bone formation, leading to extensive bone loss within two weeks of treatment [9,16]. Importantly, endogenously high EPO levels, either due to induced anemia [17] or constitutive overexpression of this hormone [9] (both in murine models), also lead to significant bone loss. In human subjects (elderly men with normal renal function) high plasma levels of EPO were associated with increased fracture risk [18]. In vitro studies have demonstrated that EPO, at concentrations between 0.1 and 5  mU/mL, has an inhibitory effect on osteoblast differentiation, while the effect of EPO on osteoclasts was stimulatory at concentrations between 10 mU/mL and 10 U/mL [9,16]. 

The aim of this study was to investigate the relationship between EPO dose, hemoglobin level and the extent of bone loss. We also explored whether the extensive loss of trabecular bone induced by transient supraphysiological levels of EPO is reversible. 

## 2. Results

**Figure 1:**
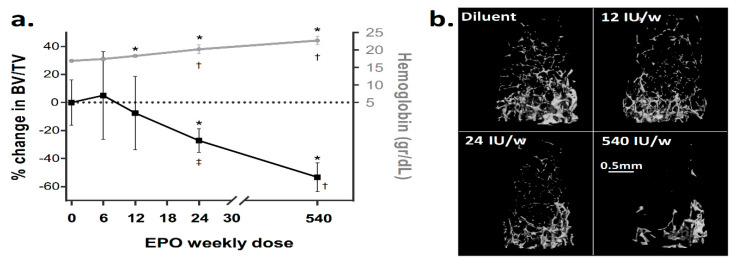
Erythropoietin (EPO) induces dose-dependent trabecular bone loss. (**a**) Relationship between the hemoglobin level (grey) and the extent of reduction in BV/TV (black) at different doses of EPO. The values on the X axis represent a total weekly dose of EPO administered to 10-week-old female mice for two weeks. Values on the Y axis are mean ± SD; *n* = 3 for 6 and 12 IU, *n* = 7 and 8 for 24 and 540 IU, respectively. (**b**) Representative 3D reconstruction images of the distal femoral metaphysis of mice treated with diluent or EPO at the designated doses. * *p* < 0.05 relative to diluent control, ^†^ *p* < 0.05 relative to preceding dose, ^‡^ *p* = 0.05 relative to preceding dose.

We have previously reported that EPO administration induces severe bone loss in mice [9]. Here, we evaluated the relationship between EPO dose, Hgb levels and the extent of EPO-induced bone loss. We treated 10-week-old female mice with a range of EPO doses for a total period of two weeks. An administered dose of 6 IU/week did not induce any noticeable hematological or skeletal effect. At a dose of 12 IU/week, we noticed a modest yet significant increase in Hgb, with no significant bone loss. In fact, our results indicated that only doses of 24 IU/week and above significantly affected the trabecular bone. At a dose of 24 IU/week we observed a 27% reduction in bone volume fraction (BV/TV, measured as the % reduction in BV/TV relative to diluent control, hereafter, ΔBV/TV). At the highest dose (540 IU/week), ΔBV/TV reached 53 ± 10.2% (Figure 1), in line with our previous findings [9]. Notably, increasing the EPO dose by 22.5 fold (from 24 to 540 IU/week) led to a modest 12% elevation in Hgb levels (20.2 ± 1.3 mg/dL vs 22.7 ± 1.3 mg/dL), whereas the ΔBV/TV dropped nearly two fold (‒27 ± 8.5% vs ‒53 ± 10.2%). This finding suggests a differential sensitivity of the erythropoietic and skeletal responses to EPO at high doses. 

**Figure 2:**
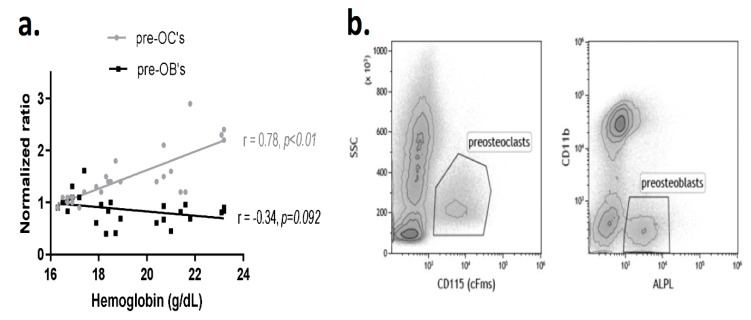
Administration of EPO is associated with a dose-dependent increase in the preosteoclast population. **a.** Linear correlation between the preosteoclast (pre-OCs, gray) and preosteoblast (pre-OBs, black) bone marrow populations versus hemoglobin level, following treatment with increasing doses of EPO as detailed in Figure 1. Values on the Y axis represent the normalized ratio of the corresponding population relative to the diluent control. Preosteoclasts were defined as CD115 (cFms)+ whereas preosteoblasts as ALPL^+^/CD11b^-^ cells in the bone marrow; **b.** Representative flow cytometry dot plots of the pre-OC (left) and pre-OB (right) populations of the EPO-treated animal (24IU/week). ALPL = alkaline phosphatase.

We also analyzed the correlation between Hgb level and the number of bone marrow preosteoclasts (pre-OCs), defined as CD115^+^ cells, as well as preosteoblasts (pre-OBs), defined as ALPL^+^/CD11b^-^ cells. The results revealed a significant positive linear correlation between the level of Hgb and the number of pre-OCs (r = 0.78, *p* < 0.01, Figure 2). There was no significant relationship between the pre-OB fraction and Hgb when considering the entire range of EPO doses (r = ‒0.34, *p* = 0.092, Figure 2). These data suggest that EPO-induced bone loss is closely correlated with the preosteoclast fraction in the bone marrow at all tested EPO doses. 

**Figure 3:**
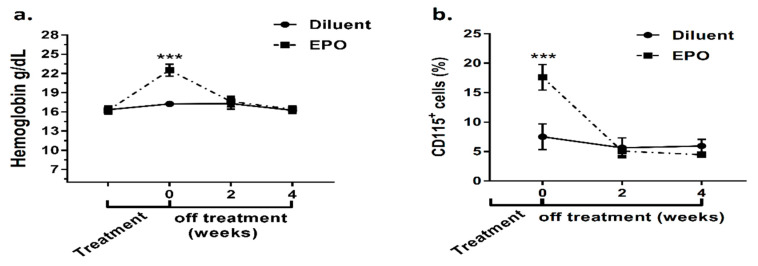
Temporal dynamics of the erythroid response and preosteoclast population following EPO stimulation. **a.** Hgb levels measured before and at the end of EPO (540 IU/week)/diluent injections (Time 0) as well as 2 and 4 weeks following treatment discontinuation; *n* = 14 for time points ‒2 and 0, *n* = 6 for other time points. **b.** Pre-osteoclast population, defined as CD115^+^ bone marrow cells, at the end of treatment, as well as 2 and 4 weeks following treatment discontinuation. Values are mean ± SD. *** *p* < 0.001; *n* = 6 for each treatment and time point.

In order to assess the reversibility of the bone changes inflicted by a surge in EPO levels, we monitored Hgb, the pre-OC fraction and bone parameters at 2-week intervals during the recovery period, i.e., after EPO discontinuation. The first measurement was done 2 weeks after the start of EPO administration and then at 2, 4, and 6 weeks after the last EPO injection. Figure 3a presents the mean Hgb levels at consecutive time points and shows that the Hgb level had already returned to baseline at two weeks post treatment discontinuation. The pre-OC population exhibited a trend similar to Hgb (Figure 3b), but bone parameters presented a more complex picture. 

**Figure 4:**
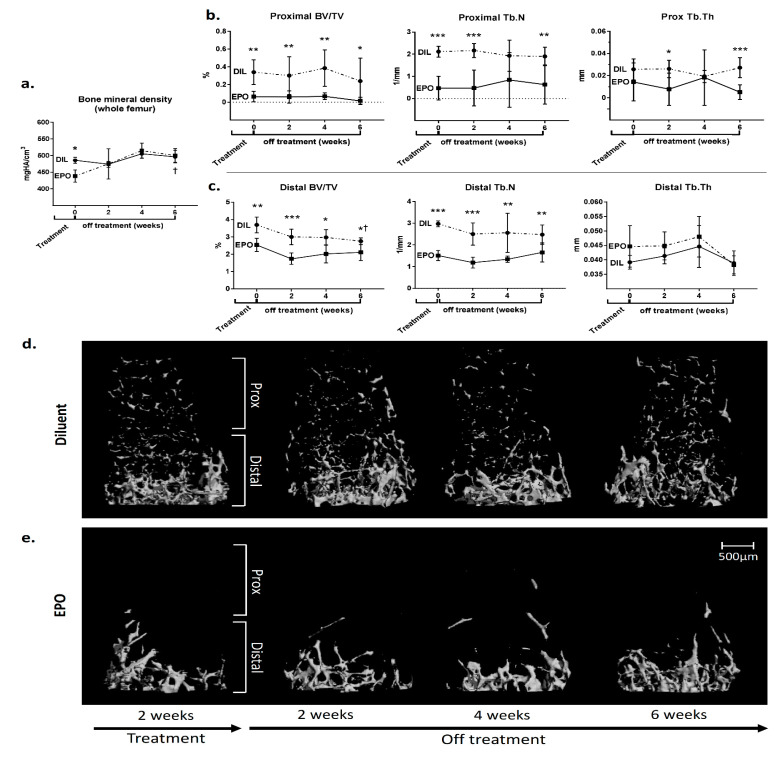
EPO induces irreversible changes in the microarchitecture of the trabecular bone of the femur. (**a**) Volumetric bone mineral density (vBMD) of the whole femur of female mice treated with either diluent or EPO (540 IU/week) at the end of the treatment period and 2, 4 and 6 weeks post treatment. (**b**,*c*) Trabecular bone volume (BV/TV), trabecular number (Tb.N) and trabecular thickness (Tb.Th) of the proximal (**b**) and distal (**c**) portions of the femoral metaphysis of mice described in (**a**). Values are mean ± SD. EPO—erythropoietin, DIL—diluent. *** *p* < 0.001; ** *p* < 0.01; * *p* < 0.05; ^†^ *p* < 0.05 relative to the “end of treatment” time point (0 weeks); (**d**,*e*) Representative 3D reconstruction images of the distal femoral metaphysis (subdivided into proximal and distal subregions) of mice treated with either diluent (**d**) or EPO (540 IU/week) (**e**) at corresponding time points. *n* = 6 for each group.

The volumetric bone mineral density (vBMD) in the whole femur returned to baseline within two weeks (Figure 4a). In the femoral metaphysis, however, the normal trabecular architecture had not fully recovered even after 6 weeks following the discontinuation of EPO (Figure 4b‒c). BV/TV and Tb.N in both the proximal and in distal parts of the distal metaphysis remained significantly lower in the EPO-treated mice even at 6 weeks after the end of treatment (Figure 4b‒c). Notably, administration of EPO for two weeks led to an almost complete and irreversible effacement of the bone trabeculae in the proximal part of the femoral metaphysis (0.238 ± 0.26% vs. 0.016 ± 0.04% in the control vs. EPO-treated mice, respectively). However, in the distal part, there was some recovery of the trabecular bone volume as reflected by the fact that the distal BV/TV of the EPO-treated mice did not change during the 6-week-long post-treatment period (2.54 ± 0.38% vs 2.11 ± 0.46% at 2- and 6-week post-treatment time points, respectively, *p* > 0.05), as opposed to the normal age-related decline in BV/TV observed in the control animals (3.69 ± 0.45% vs 2.76 ± 0.19%, respectively, *p* < 0.05). 

**Figure 5:**
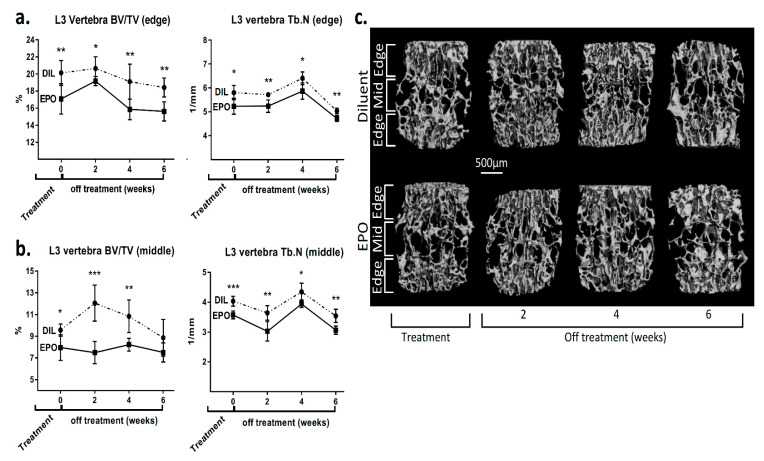
EPO induces irreversible changes in the microarchitecture of the lumbar spine vertebrae. (**a**) Trabecular bone volume (BV/TV) and trabecular number (Tb.N) of the edge (**a**) and midportion (**b**) of the L3 vertebra from diluent (DIL) and EPO (540 IU/week)-treated female mice at the completion of the treatment period and 2, 4 and 6 weeks post treatment. Values are mean ± SD. *** *p* < 0.001; ** *p* < 0.01; * *p* < 0.05; (**c**) Representative 3D reconstruction images of the L3 vertebra (subdivided into edge- and midportions) of the mice described in (**a**,**b**). *n* = 6 for each group. “Edge” and “middle” portions of the vertebral body were defined as marginal (upper/lower) and middle 1/3 of the vertebral body in the axial plane.

An irreversible loss of the trabecular bone was also noted in the spine of the experimental animals (Figure 5). At the end of the treatment period, there was a significant 15% and 17% reduction in the “edge” and “middle” BV/TV of the L3 vertebrae, respectively (Figure 5c).

At the last time point (6 weeks), the “edge” BV/TV was still 15% lower in the EPO-treated vs control mice (*p* < 0.01, Figure 5a). Although the difference in the BV/TV in the midportion of the L3 vertebral body (“middle” BV/TV) did not reach statistical significance at 6 weeks post treatment, there was still a significantly lower trabecular number in this region (4.03 ± 0.22% vs 3.58 ± 0.14% in the diluent- vs EPO-treated mice, respectively, *p* < 0.01, Figure 5b).

## 3. Discussion

Erythropoiesis, like hematopoiesis in general, is a tightly regulated process. Endogenous regulation is essential to maintain the blood elements, including red blood cells, within a predefined range, i.e., to avoid anemia (low hemoglobin levels) at one extreme and excessive erythrocytosis at the other. The former can lead to a reduction in stamina and physical capabilities, while the latter can give rise to hyper-viscosity with consequent inevitable vascular endothelial damage that could result in decreased survival and disability [19]. The regulation of erythropoiesis takes place at different levels, namely, endocrine [20], paracrine [21] and intracellular [22,23]. The endocrine regulation, mediated by erythropoietin, is the most robust and is responsible for the adaptation of the erythroid lineage to various physiological conditions, e.g., high altitude, hypoxia or anemia [24]. Beyond erythropoiesis, EPO has been shown to exert beneficial effects in neurodegenerative disorders, cardiovascular diseases and in the immune system [25,26]. Published reports by us and others have clearly demonstrated that EPO induces bone loss [9,16,17], which raises the question of whether EPO directly affects bone remodeling or is acting indirectly via increasing red blood cell (RBC) mass. In fact, several lines of evidence suggest that EPO induces bone loss directly, via its cognate receptor (EPO-R) on non-erythroid cells. First, we reported that pharmacological blockade of signaling molecules downstream of EPO-R attenuated an EPO-mediated increase in osteoclastogenesis *in vitro* [9]. In addition, the effect of low dose EPO in isolated cells was abrogated after treating cells with EPO-R-directed siRNA [16]. Finally, EPO did not affect bone mass in ΔEpoRE mice, in which EPO-R expression is restricted to erythroid cells [27].

The dose-dependent nature of the EPO-induced bone loss may stem from a differential effect of EPO on bone formation and bone resorption, which are carried out by osteoblasts and osteoclasts, respectively. Evidently, at lower EPO doses, there is a decrease in the frequency of osteoblast precursors (Figure 2a), together with an inhibition of the mineralization capacity *in vitro* [16,27], whereas at higher doses the dose-dependent increase in osteoclast precursors *in vivo* (Figure 2a) is associated with an increase in mineralization capacity shown in vitro [27]. It appears however that at higher doses the bone-resorbing effect predominates and overwhelms any possible compensatory increase in bone formation. Interestingly, the latter may explain the prompt restoration of the whole femur vBMD observed as soon as two weeks after the discontinuation of EPO treatment (Figure 4a). However, the “normalization” of the vBMD might be misleading in terms of bone health since a sizable proportion of fractures occur in patients whose BMD does not fulfill the WHO criteria for osteoporosis [28]. In this regard, it is well-established that in addition to mineral density, measures of trabecular bone microarchitecture serve as important determinants of mechanical properties and resilience to fractures, especially in weight-bearing skeletal sites that are rich in trabecular bone [29,30]. Notably, the treatment with EPO in our experiment started at the age of 10 weeks, i.e., two weeks before the peak bone mass in females [31], and ended at 12 weeks of age, i.e., at the time of the peak bone mass. This study therefore investigated the recovery from EPO treatment in young female mice prior to attaining peak bone mass, and further studies might be needed to assess whether this recovery is different in older animals. Another limitation of this study is that only anemic patients are treated with EPO, whereas the mice in our study had normal hemoglobin levels. This settings is reminiscent of the physiological changes resulting from short term periods at high altitude, characterized by an increase in EPO and Hgb levels [32] and significant bone loss [33,34]. 

An important aspect of our results is the permanent loss of trabecular architecture following EPO stimulation both in the femur and lumbar spine (Figure 4 and Figure 5). The major impact on the metaphysis was seen in the proximal part of the distal metaphysis, with an almost complete effacement of the trabeculae, which did not recover even after six weeks without treatment. This supports the notion that a profound loss of mature trabecular networks in bones formed by endochondral ossification results in the inability to rebuild new trabeculae. This is probably due to the fact that the metaphyseal trabeculae are formed as part of the growing process, and once the growth period is over, a total disappearance of trabeculae remains irreversible. Although not tested here, it is reasonable to assume that lower doses of EPO would only partially reduce the amount of the trabecular bone, preserving a sufficient backbone for a more efficient restoration of the trabecular network upon discontinuation of EPO treatment. However, our data in the vertebrae suggest that the level of recovery is independent of the remaining bone density at the end of the treatment period. Indeed, in the middle part of the vertebra, the degree of recovery was higher despite a lower bone density compared to the edges (Figure 5).

A continuing matter of debate is the initial dosing strategy in patients who require treatment with rHuEPO. Typically, patients with advanced chronic kidney disease will receive EPO at 50-100 IU/Kg three times a week, and patients with hematological malignancies (MDS, MM) will start with higher doses, i.e., 150 IU/Kg three times a week or 40,000 IU once a week. Nevertheless, some clinicians will choose to start with higher doses and then taper them down if the Hgb response is excessive (> 11 mg/dL [35]), a.k.a the “step-down” approach. Others will prefer a “step-up” approach starting with lower doses and gradually increasing the doses until the desired Hgb levels are obtained. The data presented here strongly advocate for the latter approach as the least potentially hazardous in terms of bone health. 

It should be noted that the dosing range per body mass is higher in our experimental setting than in clinical settings, i.e., ~ 100, 200, 400 IU/kg and 9,000 IU/kg per dose (see previous paragraph). However, this dosing range is most commonly used in murine studies [9,17,36] because it correlates well with the hemoglobin response. For example, administration of 4 IU per dose (200 IU/kg), which would be considered high for human patients, led to a modest increase in Hgb (from 16.9 ± 0.29 to 18.3 ± 0.15 mg/dL, *p* < 0.05), whereas the lowest dose of 2 IU (100 IU/kg) did not increase the hemoglobin levels (Figure 1). Notably, recombinant human EPO was never tested in comparission to rodent EPO and the affinity of human EPO may be different to that of murine EPO. Indeed, a study conducted in dogs reported significant differences between the erythroid response to the canine EPO versus its human homologue [37].

It is also interesting to note that the dose-response relationship between EPO, Hgb response and bone loss, especially at higher doses, indicates the presence of differences between the magnitude of the erythropoietic as compared to the skeletal effects of EPO. As seen in Figure 1a, administration of EPO in doses ranging from 24 to 540 IU/week led to a modest 12% increase in hemoglobin, while bone tissue exhibited an almost two-fold reduction in trabecular bone volume. One molecular mechanism that may at least partially account for this phenomenon is the opposite effect of the suppressor of cytokine stimulation (SOCS) 1 and 3 on erythropoiesis and osteoclastogenesis. In erythroid cells, SOCS1 and SOCS3 inhibit EPO signaling by interacting with JAK2 [23,38], while in preosteoclasts, EPO-mediated expression of SOCSs counteracts the inhibitory action of interferon β (IFNβ) induced by RANKL as part of an autocrine inhibitory loop [39]. 

In conclusion, our results complement previous reports by showing that EPO induces a dose-dependent and mostly irreversible bone loss. Since EPO is widely used in clinical practice, especially in patients who are frequently prone to osteoporosis, further studies are warranted to explore the skeletal effects of this intervention in human patients. Nevertheless, until sufficient human data become available, it is reasonable to favor the lowest effective dose of EPO for the shortest period of time. Such an approach would not only decrease the risk of thromboembolic complications but also minimize the potential for adverse skeletal outcomes. 

## 4. Methods

### 4.1. Experimental Animals 

For the dose-response experiments, C57BL/6J-RccHsd (Envigo, Jerusalem, Israel) 10-week-old female mice were injected three times a week, for two weeks with either diluent or recombinant human erythropoietin (Epoetin α, Eprex®, Janssen) (hereafter, EPO), at doses of: 6, 12, 24 or 540 IU/week. Animals were housed in the SPF animal care facility with a 12-hour light/dark cycle and fed a normal chow diet. All experiments were performed in accordance with the Institutional Animal Care and Use Committee of Tel-Aviv University. 

For the recovery experiment, 4 groups of C57BL/6J-Hsd-RCC 10-week-old female mice were injected three times a week for two weeks with EPO (540 IU/week) and 4 groups were injected with diluent. The animals in one group from each treatment arm were euthanized at the end of the 2-week treatment, and the other groups were maintained with no further treatment for 2, 4 and 6 weeks. The C57BL/6J-RccHsd mice historically originated from the C57BL/6J (Jackson Laboratory, Bar Harbor, Maine) and were moved in 1973 to Europe where they naturally developed a genetic drift.

### 4.2. Microcomputed Tomography (µCT)

Femora (1 per mouse) and the L3 vertebra were examined using a µCT50 system (Scanco Medical AG, Switzerland). Briefly, all scans were performed at a 10 µm resolution, with 90 kV energy, 88 µAmp intensity and 1000 projections with 1100 ms integration time. The mineralized tissues were segmented using a global thresholding procedure, using a value of 160 permille of maximal gray values (corresponding to 504 mgHA/cm^3^) for the trabecular bone.

Trabecular bone parameters were measured in a 3 mm height volume in the secondary spongiosa of the distal femoral metaphysis ending distally at the limit of the primary spongiosa. The secondary spongiosa in the distal femur was then divided into a proximal and a distal halves. We also measured the volumetric bone mineral density (vBMD) in the entire femur. In the L3 vertebra, the trabecular bone was evaluated in the entire volume of the vertebral body, excluding the primary spongiosa at both ends. In L3, the trabecular bone was divided in 3 caudo-rostral (axial) thirds, in order to discriminate between the second (middle) compartment and the first and third volumes (edges). All morphometric parameters were evaluated and presented according to the standardized nomenclature [40].

### 4.3. Hemoglobin Levels

Hgb levels were measured in venous blood (drawn from the facial vein) by means of a “Mission Plus” hemoglobin/hematocrit meter (Acon, San Diego, CA, USA).

### 4.4. Flow Cytometry

Bone marrow (BM) cells were flushed from either femur or tibia. Red blood cells were lysed using ACK lysis buffer (Quality Biological, Gaithersburg, MD). BM cells were stained for 30 min at 4 °C with conjugated anti-mouse antibodies: CD115-allophycocyanin (APC), CD11b-Pacific Blue™ or CD11b-FITC. For alkaline phosphatase (ALPL) staining, we used primary goat anti-mouse ALPL followed by PE-conjugated donkey anti-goat secondary antibody (R&D, Minneapolis, MN, USA) or APC-conjugated donkey anti-goat secondary antibody (AnaSpec, Fremont, CA, USA). 

### 4.5. Statistical Analysis

Results are presented as means ± standard deviation (SD) (except in Figure 2, where the Pearson correlation is presented). Either Student’s *t*-test or nonparametric (Mann-Whitney U) test for tstatistical significance were used to calculate *p* values for samples with n≥5 and n<5, respectively. Statistical significance was determined when *p* < 0.05. GraphPad Prism v7.04 software was used for statistical analyses.
